# Pharmacological Features and Therapeutic Implications of Plumbagin in Cancer and Metabolic Disorders: A Narrative Review

**DOI:** 10.3390/nu16173033

**Published:** 2024-09-08

**Authors:** Bhoomika Sharma, Chitra Dhiman, Gulam Mustafa Hasan, Anas Shamsi, Md. Imtiyaz Hassan

**Affiliations:** 1 Department of Biosciences, Jamia Millia Islamia, Jamia Nagar, New Delhi 110025, India; bhoomika124@gmail.com (B.S.); dchitra1103@gmail.com (C.D.); 2 Department of Basic Medical Science, College of Medicine, Prince Sattam Bin Abdulaziz University, Al-Kharj 11942, Saudi Arabia; mgulam@jmi.ac.in; 3 Centre of Medical and Bio-Allied Health Sciences Research, Ajman University, Ajman P.O. Box 346, United Arab Emirates; 4 Centre for Interdisciplinary Research in Basic Sciences, Jamia Millia Islamia, Jamia Nagar, New Delhi 110025, India

**Keywords:** plumbagin, natural products, anticancer, anti-inflammatory, apoptosis, antioxidant, cardiovascular disease, neurodegenerative diseases

## Abstract

Plumbagin (PLB) is a naphthoquinone extracted from *Plumbago indica*. In recent times, there has been a growing body of evidence suggesting the potential importance of naphthoquinones, both natural and artificial, in the pharmacological world. Numerous studies have indicated that PLB plays a vital role in combating cancers and other disorders. There is substantial evidence indicating that PLB may have a significant role in the treatment of breast cancer, brain tumours, lung cancer, hepatocellular carcinoma, and other conditions. Moreover, its potent anti-oxidant and anti-inflammatory properties offer promising avenues for the treatment of neurodegenerative and cardiovascular diseases. A number of studies have identified various pathways that may be responsible for the therapeutic efficacy of PLB. These include cell cycle regulation, apoptotic pathways, ROS induction pathways, inflammatory pathways, and signal transduction pathways such as PI3K/AKT/mTOR, STAT3/PLK1/AKT, and others. This review aims to provide a comprehensive analysis of the diverse pharmacological roles of PLB, examining the mechanisms through which it operates and exploring its potential applications in various medical conditions. In addition, we have conducted a review of the various formulations that have been reported in the literature with the objective of enhancing the efficacy of the compound. However, the majority of the reviewed data are based on in vitro and in vivo studies. To gain a comprehensive understanding of the safety and efficacy of PLB in humans and to ascertain its potential integration into therapeutic regimens for cancer and chronic diseases, rigorous clinical trials are essential. Finally, by synthesizing current research and identifying gaps in knowledge, this review seeks to enhance our understanding of PLB and its therapeutic prospects, paving the way for future studies and clinical applications.

## 1. Introduction

Plants synthesize complex chemical compounds known as plant secondary metabolites in response to specific biotic and abiotic stresses [1]. In addition to protecting the plant, secondary metabolites are essential for a plant’s physiological tasks, such as pollination, vascular development of lignified walls, etc. The ability of these metabolites to elicit a toxicological response in humans has made these compounds the centre of attention for the pharmaceutical industry [2]. 

Quinones are a type of secondary metabolite synthesized in plants and are named after their quinone form [3]. Based on their benzene rings, quinones are primarily classified into four different categories: phenanthrenequinone, anthraquinone, benzoquinone, and naphthoquinone. Recently, quinones have attracted considerable interest among pharmacological enthusiasts, largely due to their therapeutic potential against various diseases [4].

Naphthoquinones, naturally occurring in plants, have been used in human life since ancient times [5]. More and more studies elucidating the role of natural and artificial naphthoquinones have realized their pharmacological and biological significance. The most widely documented and stable isoform is known as 1,4-naphthoquinone. Various analogues have been discovered based on chemical modifications of 1,4-naphthoquinone, such as Juglone, Plumbagin, Shikonin, anthraquinone, etc. [6]. 

One of the analogues, Plumbagin (PLB), is isolated from the families *Ebenceae*, *Plumbaginaceae*, and *Droseraceae* [7]. PLB, 2-methyl l-5-hydroxy-1,4-naphthoquinone, is a Vitamin K3 analogue [8]. It was initially isolated from *Plumbago indica* and is a principal component in the roots of Plumbago *zeylanica*, also designated as Chitrak in Ayurvedic medicine. Additionally, it is frequently observed in the carnivorous plant genera *Drosera* and *Nepenthes* and constitutes a constituent of the black walnut drupe [9]. 

Many studies have demonstrated numerous beneficial properties of PLB, including antioxidant, antidiabetic, antifungal, and, most notably, anticancer effects. Recent data have shown that PLB exerts an inhibitory effect on cancer progression in a range of cancer cell lines [10,11]. It has been shown that important pathways involved in cancer progression, such as the Akt/NF-kB signalling pathway and the MMP-9 pathway, are prime targets involved in the PLB-mediated cancer response. 

In addition, activation of Reactive Oxygen Species (ROS), cell cycle arrest, and apoptotic signalling are crucial for PLB-induced anticancer response [12,13]. In Lewis Lung carcinoma (LLC) lung cancer cells, evidence suggests that PLB interactions with Thioredoxin Reductase (TrxR) prevent its downstream interactions with intracellular substrates and directly inhibit Glutathione Reductase (GR), thereby mediating the increase of intracellular ROS levels and ultimately leading to apoptosis [12].

Furthermore, the compound demonstrated considerable inhibitory effects via the IL-6/STAT3 signalling pathway, thereby impeding the proliferation and invasion of lung cancer cells [14]. PLB exerts its effect via various signalling mechanisms. For instance, PLB upregulates the expression levels of p21^CIP1/WAF1^, causing the cell cycle to arrest in the G2/M phase, thereby inducing apoptosis [15]. The administration of PLB has been demonstrated to result in the downregulation of cytokine expression and the suppression of NF-κB-regulated genes in both MM-231 and MM-468 triple-negative breast cancer cell lines [16]. 

Besides cancer, it has been reported that PLB possesses anti-viral properties against RNA viruses such as Hepatitis C Virus (HCV), as well as SARS-CoV-2, due to its ability to generate ROS-mediated oxidative stress against their highly susceptible single-stranded RNA genome [17,18]. Researchers have demonstrated the anti-inflammatory role of PLB in neurodegenerative diseases such as Parkinson’s disease, where PLB could effectively provide neuroprotection to PD mice models by inhibiting inflammation via the TLR/NF-κB pathway and reduced expression of IL-1β, TNF-α, and IL-6 mRNA levels [19]. Its role is also evident in cardiovascular diseases, as PLB showed a significant drop in the blood pressure of the rats studied. In addition, PLB has an antibacterial effect on the organism, effectively limiting its growth [20,21]. 

**Figure 1:**
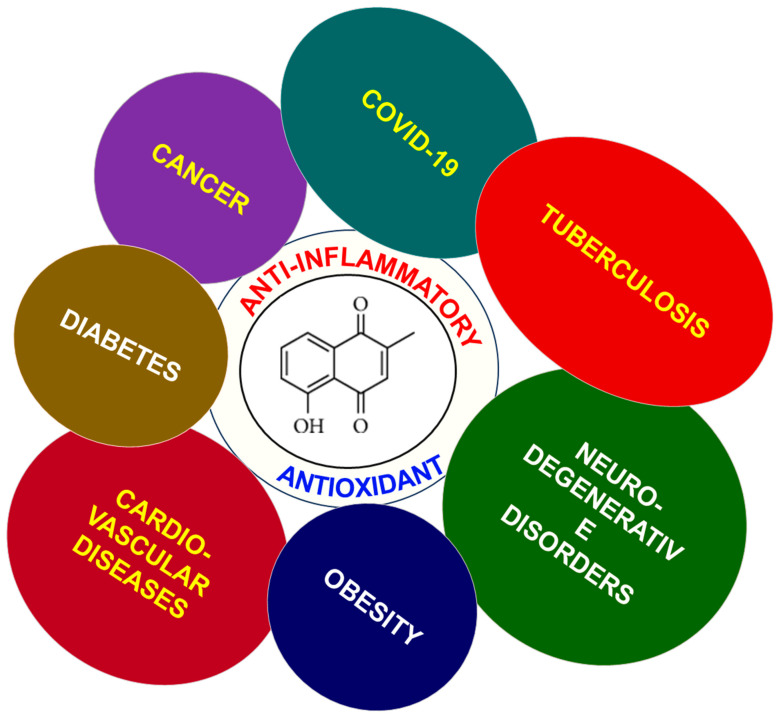
Structure of PLB with its role in preventing various diseases.

In an attempt to address the vast spectrum of therapeutic properties of PLB, this review article summarizes different pharmacological attributes, such as chemical properties and the biosynthesis of PLB. It also highlights the importance of PLB as an emerging therapeutic candidate, as documented in the literature for various diseases such as cancer, SARS-CoV-2, diabetes, cardiovascular diseases, etc., as their prevalence has significantly impacted the lives of millions and has resulted in the loss of lives (Figure 1). 

We further discuss the clinical formulations reported for PLB to increase its efficacy, which will further aid in its use as a potential drug and eventually support the healthcare system. In a nutshell, this review reflects on the potential therapeutic benefits and uses of PLB in the care and management of cancer, as well as other diseases.

## 2. Methods

For this review, a systematic literature search was conducted using several major databases, including PubMed, Google Scholar, and Science Direct. The search utilized specific keywords such as “Plumbagin”, “cancer”, “disorders”, and “bioavailability” to identify relevant studies. As of 2024, a total of 337 articles were retrieved from these databases. Each article was carefully evaluated for relevance to the scope of the review. Following a thorough assessment, 200 articles were deemed pertinent and included in the review. This selection process ensured that the review encompasses a broad and relevant range of research on the therapeutic potential of PLB and its pharmacological properties.

## 3. Chemical Properties of PLB

PLB is a non-chiral, lipophilic compound with a molecular mass of 188.18 g/mol [22]. It exists in the form of a yellow crystalline substance. Experimentally, it is soluble to 100 mM in DMSO and to 50 mM in ethanol. Since it is sparingly soluble in water, its clinical translation requires formulations such as nanoemulsions [23,24]. The basic skeleton of the naphthoquinone is produced by a Polyketide Synthase (PKS) using six acetyl units [25] with 3-methyl-1,8-naphthalene-diol as an intermediary product. The PLB biosynthetic pathway also involves certain accessory enzymes, such as cyclase1 and AKR1 [26].

PLB is a part of the human exposome, having a role as a metabolite, an immunological adjuvant, an anticoagulant, and an antineoplastic agent. Predictions from the Swiss Absorption, Distribution, Metabolism, and Excretion tool (SwissADME) which suggested many drug-like features (http://www.swissadme.ch/ last accessed date: 8 August 2024). It has a positive LogP value of 1.72, which follows Lipinski’s rule of 5 for drugs. PLB-labelled complex was found to have a short elimination half-life of about 2–3 h in blood. The excretion of PLB is primarily via the hepatobiliary and pulmonary routes, with limited pharmacokinetic properties resulting in inadequate systemic circulation. [27]. Nanoencapsulation of PLB overcomes the obstacles of low water, poor water-dissolving ability, and bioavailability, adding to pharmaceutical relevance with better therapeutic efficacy [28]. Important chemical and drug-like features of PLB are highlighted in Table 1. 

## 4. Medicinal Properties of PLB

Ayurveda, a traditional system of medicine native to India, uses plant extracts, including PLB, to treat various diseases in the country [29]. However, it gained the attention of scientists worldwide only a few years ago, owing to the growing chemo-drug resistance in patients. Studies have shown that PLB possesses antifungal, antiviral, antibacterial, and antioxidant properties, as well as other biological properties such as anti-diabetic, analgesic, and anti-atherosclerosis [10]. It is widely established that inflammation represents a fundamental causal factor in a considerable number of pathological conditions, including cancer. PLB has been demonstrated to show strong anti-inflammatory properties, making it an interesting research candidate for treating cancer [30,31].

### 4.1. Role of PLB in Cancer Therapy

**Figure 2:**
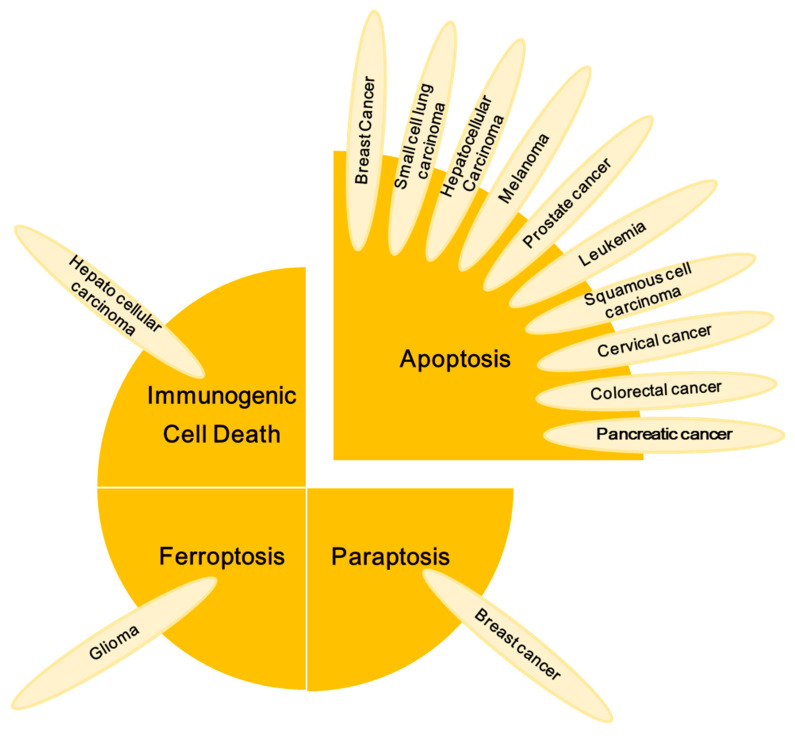
Modes of action of PLB in the treatment of various forms of Cancers. While apoptosis is the primary pathway involved in restricting cancer growth, other pathways, such as immunogenic cell death, ferroptosis, and proptosis, are also involved.

PLB has emerged as a promising compound in cancer therapy, due to its multifaceted therapeutic effects. The cytotoxicity of PLB against cancer cells has been observed in both in vitro and in vivo settings [28]. Anticancer activity of PLB has been reported against various cancer cell lines, including hepatoma, melanoma, leukaemia, breast carcinoma, prostate cancer, oral squamous-cell carcinoma, brain cancer, oesophageal cancer, lung cancer, kidney adenocarcinoma, cholangiocarcinoma, osteosarcoma, gastric cancer, and canine cancer [10]. The activity of PLB against cancer cells is summarized in Figure 2 and Table 2.

**Figure 3:**
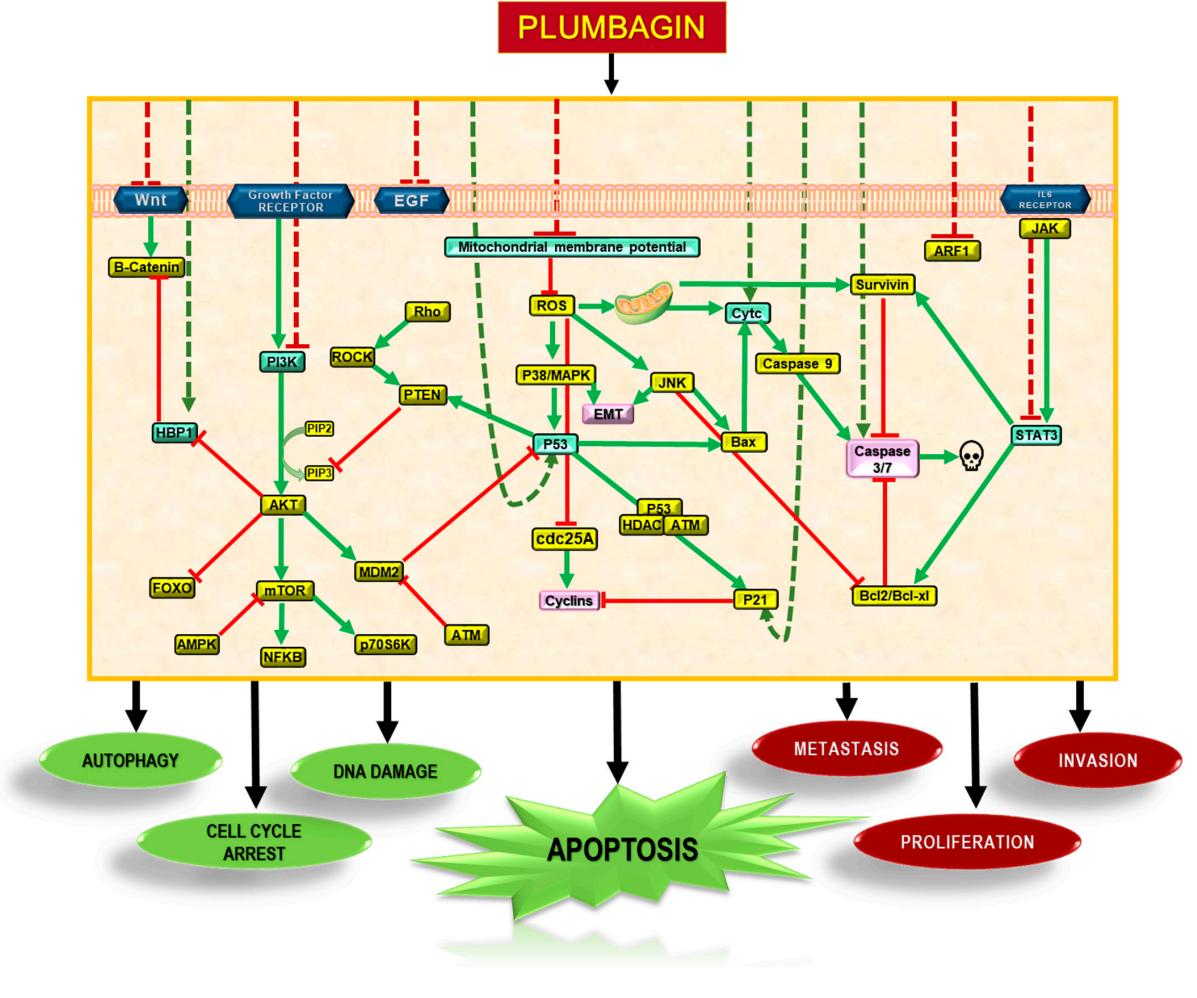
Molecular mechanisms of action of PLB. PLB downregulates several pathways in tumour cells to induce autophagy, cell cycle arrest, and DNA damage. These mechanisms inhibit metastasis and proliferation via Akt/PI3K, STAT3, NF-κB, and Wnt pathways. PLB introduces a cascade of signalling pathways that eventually stimulate apoptosis in cancerous cells.

Numerous studies reported on the mechanism of the anticancer activity of PLB, which is attributed to its ability to exert an effect through different signal transduction pathways, including those involving PI3K/AKT/mTOR, Ras, Sirt1, AMPK, CDK1/CDC2, cyclin B1, cyclin D1, FOXM1, NF-κB, Nrf2/ARE, p53, p21 Waf1/Cip1, p27 Kip1, PI-5, STAT3/PLK1/AKT, and Wnt. Therefore, PLB may be a promising candidate for further investigation as a potential therapeutic agent for cancer treatment [22,41]. PLB has been shown to exert its effects primarily through the PI3/AKT/mTOR pathway in cancer cells, as illustrated in Figure 3. Self-regulated processes such as epithelial-to-mesenchymal transition (EMT) required for tissue repair may lead to conditions such as angiogenesis, fibrosis, loss of normal organ function, and even cancer in an uncontrolled state [42]. 

#### 4.1.1. Breast Cancer

Breast cancer, a leading cause of cancer-related deaths among women worldwide [43], is driven by dysregulated pathways such as cell cycle, apoptosis, Wnt-signalling, DNA damage-repair pathways, inflammation, and hypoxia. Inflammation, in particular, is a known modulator of the progression and aggressive growth of breast cancer [44]. The NF-κB gene, a well-established regulator of the inflammatory response, plays a significant role in this context. Overstimulation of the NF-κB gene is a contributing factor to the aggressive nature of breast cancer [45]. One of the cytokines released in the inflammatory pathway of NF-κB is the C-C motif ligand, also known as CCL-2. CCL-2 is a chemokine that attracts immune cells to the site of inflammation and is purported to be implicated in most types of cancer, making it a crucial target for therapeutic intervention [46]. Endocrine therapy has shown promising avenues of treatment in early-stage breast cancer; however, advanced-stage breast cancer cells show poor response due to their increased chemoresistance [47]. Furthermore, the absence of the three essential endocrine receptors, progesterone (PR), oestrogen (ER), and human epidermal growth factor (Her2) receptors in triple-negative breast cancer (TNBC) cells significantly diminishes the efficacy of the treatment. Thus, studies for PLB for targeting inflammatory factors and cytokines have shown this secondary metabolite to be implicated in controlling breast cancer progression [47]. 

PLB downregulates the expression of CCL2 cytokine and suppresses NF-κB-regulated genes in TNBC cell lines MM-231 and MM-468 [16]. The inhibitory effect of PLB on breast cancer progression in human endocrine-resistant cells (MCF-7/LCC2 and MCF-7/LCC9) is particularly pronounced, given that approximately half of breast cancer patients with advanced-stage ER-positive breast cancer develop endocrine resistance [48]. PLB targets the AKT signalling pathway and inhibits the progression by inhibiting AKT (pAKT) and pERK1/2 phosphorylation, regulated through Nuclear Receptor Coactivator 3 (NCOA3) in HER2-overexpressed endocrine-resistant cells. NCOA3 is a chromatin remodeler and a transcriptional factor involved in the progression of different cancers [49].

A further significant factor contributing to the growth of cancer is hypoxia. Due to the rapid development of cancer cells, the demand-supply equilibrium of oxygen is significantly disturbed, creating a hypoxic intra-tumour environment [50,51]. However, it is found that the cancer cells have adapted to maintain the equilibrium by increasing the expression of a transcriptional factor known as Hypoxia Inducible Factor (HIF-1α), which is responsible for preserving oxygen homeostasis in the body. But, in the case of cancer, it creates an environment conducive to the growth of tumour cells, resulting in rapid proliferation and, eventually, metastasis [51,52]. HIF-1α also upregulates important growth regulatory pathways such as AKT and ERK pathways. These further help tumour cells maintain growth, interfering with the cell’s natural response of apoptosis, thus developing resistance against therapeutic agents. PLB downregulates the expression of HIF-1α under hypoxic conditions in MCF-7 cells via a pathway independent of PI3K/Akt/mTOR by abolishing HIF-1α at both the transcription and post-translational modification levels [53]. 

Among many cell signalling pathways reported through which PLB has been reported to exert its anticancer effect, one is mediated through p53, an anti-tumour molecule. A recent study has demonstrated a correlation between cell cycle regulators and PLB [15]. They reported that PLB upregulates p21^CIP1/WAF1^, causing activation of apoptosis in a p53-dependent pathway, as well as the cell cycle, to arrest the G2/M phase via inhibition of cyclin B1 levels. PLB also down-regulates the anti-apoptotic genes pro-caspase 3 and Bcl-2 [54].

One of the most significant pathways in cancer treatment is the apoptosis of tumour cells. Nevertheless, cancer cells have evolved to successfully escape the event of apoptosis, leading to drug resistance [55]. Fortunately, PLB showed therapeutic potential for apoptosis-resistant cancers, i.e., PLB-induced paraptosis. This was evidenced by a trigger of extensive cytoplasmic vacuolation, which subsequently resulted in cell death. Notably, this process was neither apoptotic nor autophagic. Paraptosis is a caspase-dependent cell death pathway that involves cytoplasmic vacuolation caused by mitochondrial swelling and endoplasmic reticulum (ER) dilation [56]. Tumour cells actively synthesize new proteins to support their growth and proliferation. PLB has been demonstrated to induce cell death in such cells via a protein synthesis-dependent mechanism [55]. PLB reacts with the free thiol groups present on newly synthesized proteins, generating misfolded proteins and disrupting sulfhydryl homeostasis. This eventually generates a stress signal, resulting in ER-associated degradation (ERAD) and a subsequent loss of mitochondrial membrane potential (MMP), ultimately resulting in cell death [57,58].

Another noteworthy attribute of PLB is its ability to re-sensitize the chemo-resistant cancer cells, either in conjunction with other agents or alone [28]. It is established that a major limitation in using chemotherapy for cancer is the capacity of cells to develop resistance to these drugs. This acquired resistance is attributed to the MAPK/ERK pathway, which is a pro-survival pathway comprising a series of protein kinases, including BRAF and MEK, among others [59]. Once activated, these ERK protein kinases phosphorylate apoptotic regulators, leading to continued cell proliferation [60]. Paclitaxel is an anti-microtubule drug that inhibits tumour cell growth by causing tubulin dimerization and inhibiting microtubule depolymerization, thus preventing mitosis. It induces apoptosis independent of ERK signalling, but shows an affinity for GFs such as ERK and Ras, as they can co-localize with microtubules [61,62]. 

PLB reduces p-ERK levels and re-sensitizes paclitaxel-resistant breast cancer cells to paclitaxel-inducing cell death [63]. In a similar study, PLB successfully restored the sensitivity of breast cancer cells to the drug tamoxifen. Tamoxifen is an early-stage anticancer drug. It is a selective oestrogen receptor modulator [64]. Increased expression of an hsp70 class chaperone, GRP78, in tumour cells, reduces Bik’s expression, a pro-apoptotic protein, eventually resulting in the development of drug resistance in cancer cells [65,66]. A total of 50% of advanced-stage cancer patients develop acquired resistance to the drug Tamoxifen [67]. PLB restores the sensitivity to Tamoxifen by inhibiting the anti-apoptotic activity of GRP78, thereby inducing Bik expression and rendering cancer cells susceptible to apoptosis. PLB also enhances sensitivity of endocrine-resistant breast cancer cells for Tamoxifen by suppressing the epithelial-to-mesenchymal transition (EMT) process, a crucial stage in the metastatic spread of cancer cells. Furthermore, it was reported that transcription factors are overexpressed at both transcriptional and protein levels, thereby triggering EMT. PLB restores the CDH1 expression levels by regulating the expression of snails, leading to reduced tumour progression in these patients [68,69,70]. A synergistic effect of PLB with paclitaxel and Tamoxifen was also observed [63].

All these studies suggest that PLB is a potent therapeutic agent for cancer treatment. However, studies have also shown that PLB treatment alone lacks cell specificity [71,72], and its weak lipophilic nature results in reduced intercellular uptake [73]. 

#### 4.1.2. Lung Cancer

Lung cancer represents a significant global health burden, accounting for a considerable proportion of cancer-related mortalities [74]. Primarily caused by smoking and tobacco product usage, lung cancer can be categorized into two main histological types: small-cell lung carcinoma (SCLC) and non-small-cell lung carcinoma (NSCLC). Among these, NSCLC is the most frequently diagnosed form of lung cancer type and accounts for a significant proportion of lung cancer-related fatalities [75]. Targeted therapy is the most preferred therapy, with a good prognosis. However, with an increased risk of developing drug resistance, the therapy poses reduced therapeutic efficacy. Other treatments, such as chemotherapy, are associated with a risk of adverse reactions and harsh side effects [76]. With phytotherapeutic properties, PLB represents a promising candidate for treating patients affected with lung cancer. It works by targeting intrinsic mitochondrial apoptotic pathways and ROS generation [77]. 

A study on H195 and H160 cell populations shows the potent antitumor activity of PLB against NSCLC in a dosage-dependent manner. This is achieved by elevating ROS, Ca^2+^, and CD8+T cells and downregulating ADP-ribosylation factor 1 (ARF1), which is a key target of PLB [78]. ARF1 belongs to a class of Ras-related GTP-binding proteins. Its overexpression is associated with a poor cancer prognosis [79]. Another experiment demonstrated that the elimination of ARF1 disrupts lipid metabolism, resulting in the aggregation of lipid droplets and the stimulation of an immune response. It was also observed to cause mitochondrial disruption, leading to an elevated ER stress response, inducing CD8+T cells in the circulation, a possible mechanism used by PLB to restrict cancer cell proliferation [78]. Mitochondrial apoptotic pathway-mediated cell death is important in eradicating cancer cells. A pivotal compound in this process is Caspase 9. 

PLB treatment is found to upregulate Bax protein, facilitating the release of Caspase 9 via Cytochrome *c* (Cyt-*c*). Once released in the cytosol, it induces the expression of Apoptosis protease activating factor 1 (APAF1), which subsequently interacts with the apoptosis-associated factor 15 (Apaf-1) to create the apoptosome. The apoptosome activates caspase 9, which activates Caspase 3 downstream. Thus, PLB upregulates the expression levels of cytochrome c and induces caspase-9 and caspase-3 activity in NSCLC, ultimately resulting in the apoptosis of A549 cells [77,80]. In normal cells, PLB prevents radiation-induced apoptosis by inhibiting caspase-3 activity [81].

Matrix Metalloproteases (MMPs) promote tumour growth via matrix barrier degradation, and facilitate angiogenesis. PLB reduces MMP expression via increased ROS production. It has been demonstrated that PLB treatment results in the increased expression of antioxidant genes, specifically GSTP1 and SOD2, due to its intrinsic oxidative stress-inducing properties. The same study on A549 and NCI-H522 found that PLB treatment induces apoptosis at lower concentrations (0–6 µM). Change in morphologies of cancerous cells, including shrinkage of the cells and plate detachment, was depicted in tumour spheroid models. Following PLB treatment, A549, and NCI-H522 cells also demonstrated reduced single-cell colony-forming ability, along with reduced migration. The study showed that PLB restricts cell proliferation through cell cycle arrest, like other cancers. PLB inhibited A549 cells in the G2/M and S phases, whereas NCI-H522 cells were limited to the G2/M phase [82]. It was also noted that in A549 cells, PLB restricts the cell cycle by regulating the expression of cdc2 and cyclin B1, both promoting cell cycle progression through the G2/M phase. 

PLB induces autophagy in lung cancer cell lines A549 and H23 via inhibition of the PI3K/Akt/mTOR pathway. PLB demonstrated pro-apoptotic and pro-autophagic properties in a dose-dependent manner. The study also shows that PLB could perform apoptosis via p53-dependent and p-53-independent pathways, as H23 cells are p53 mutant cell lines. A concentration-dependent increase in the expression of PUMA, a pro-apoptotic protein, was observed in A549 cells. However, it was not replicated in the H23 cell line. Elevated expression levels of PUMA inhibit anti-apoptotic proteins like Bcl2 and Bcl-xl, thereby promoting Bax- and Cas9-mediated apoptosis [83]. Even though PLB initiates programmed cell death in cancer cells via both apoptosis and autophagy, working in a coordinated manner, the latter plays a negligible role in determining the effectiveness of PLB [82]. In the A549 lung cancer cell line, PLB also initiates paraptosis via proteasome inhibition and disruption of sulfhydryl homeostasis [57]. 

PLB inhibits the Rho-associated kinase (ROCK) pathway via the FAK/AKT pathway, thereby suppressing lung metastasis in A549 cells treated with osteopontin (OPN) [13]. OPN is a tumour-microenvironment (TME) component that is actively secreted by tumour cells and plays a pivotal role in the progression of cancer cells by upregulating MMP2/9, VEGF, and other key factors. OPN actively phosphorylates FAK and AKT and induces the ROCK pathway [84]. PLB interacts with TrxR and GR in LLC lung cancer cells via intracellular ROS [14]. Furthermore, PLB considerably inhibits the proliferation and invasive potential of L9981 and NL9980 cells via the IL-6/STAT3 signalling pathway [14].

Despite being a common treatment for cancer, the collateral damage associated with radiation therapy requires attention to finding safer and less cytotoxic alternatives like phytochemicals. PLB, when given in combination with radiotherapy, resulted in a less cytotoxic response in normal cells [85]. Thus, it showed that PLB provides radioprotection to non-cancerous cells; however, it did not render any such protection to tumour cells, therefore increasing the efficacy of radiation therapy. 

#### 4.1.3. Hepatocellular Carcinoma

Hepatocellular carcinoma represents the most prevalent form of liver cancer, and is the fourth most commonly diagnosed neoplasm worldwide [86]. PLB inhibits hepatocellular cancer progression [87]. PLB induces ATM-p53 pathway-mediated G2/M cell cycle arrest and downregulation of 25C (cdc25C), resulting from ROS-mediated oxidative stress [88]. PLB inhibits the growth of HCC cells by downregulating Glutathione peroxidase 4 (GPX4) via its ubiquitination and resultant proteasomal degradation. GPX4 protects the cells from lipid peroxidation by reducing lipid peroxides, thus maintaining cell homeostasis [89]. It negatively regulates oxidative stress-induced apoptosis. Among different antioxidant enzymes studied, such as SOD1, CAT, and TXN, PLB showed selectivity toward GPX4. GPX4 has been identified as a novel substrate of USP31, a deubiquitinase (DUB) that plays a crucial role in regulating cell proliferation. Inhibition of USP31 via PLB makes GPX4 available for protein degradation, followed by tumour cell apoptosis. Contrary to other tumours, where USP31 is shown to act as a tumour suppressor, its inhibition by PLB in HCC showed reduced cancer progression [90].

PLB inhibits cell proliferation by preventing angiogenesis in HCC cell line SMMC-7721 in a dosage- and time-dependent manner. Stromal cell-derived factor (SDF1), a chemokine, promotes angiogenesis via VEGF/IL-8 mediated NF-kb activation. In this study, PLB abolishes SDF-1-induced tumour progression by inhibiting the expression of CXCR4 and CXCR7, transmembrane receptors of SDF-1 [91]. PLB also upregulates the caspase-3 protein level in the same cell line and causes cell apoptosis via signal-mediated EMT [92]. PLB inhibits proliferation by inhibiting the SIVA/mTOR signalling pathway in HCC cell lines HepG2 and LM3, resulting in apoptosis [87]. SIVA is an apoptosis regulator protein known to promote tumorigenesis via the mTOR pathway. The signalling pathway is crucial in cell proliferation and cycle progression. Therefore, PLB’s ability to restrict tumour progression through multiple signalling pathways makes it a key therapeutic candidate for treating HCC. However, attempts have been made to enhance the activity of PLB in cases of immune resistance, including PLB-mediated immunogenic cell death (ICD). Nano co-delivery of PLB, along with Dihydrotanshinone (DIH), a natural compound from the roots of *Saliva miltiorrhiza*, helps overcome immunotherapy resistance caused by TME in HCC. PLB is an ICD inducer and Dihydrotanshinone ICD enhancer [93].

#### 4.1.4. Melanoma

Melanoma is known as the most dangerous form of skin cancer, exhibiting high drug resistance and poor prognosis [94,95]. PLB exhibited a dose-dependent reduction in cell viability among melanoma cell lines [96]. In SK-MEL-28 cells, PLB induces metabolic alterations by inducing Mitochondrial Oxidative Phosphorylation (OXPHOS). In PLB-induced A375 cells, mitochondrial OXPHOS and ATP production levels were decreased, whereas Mitochondrial Membrane Potential (MMP) was elevated. PLB augmented the generation of ROS and increased proton leak in both cell lines [97]. In B16F10 melanoma cancer cells, PLB downregulates genes associated with the MAPK pathway, MMPs, and cell adhesion, whereas it elevates the expression levels of apoptotic, ROS response, and tumour suppressor genes, suggesting an anti-invasive and anti-metastatic role of PLB [94]. Furthermore, a combination of PLB and the antineoplastic drug celecoxib has been demonstrated to exert a synergistic inhibitory effect. This was evidenced by a reduction in the proliferation of melanoma cells and the suppression of vascular development in tumours, mediated by COX-2 and STAT3 inhibition. This subsequently led to a decrease in the levels of the key cyclins that are essential for melanoma cell survival [95].

#### 4.1.5. Prostate Cancer

Prostate cancer represents the second most prevalent form of cancer diagnosed in males worldwide [98]. These cancers are most commonly adenocarcinomas. Currently, both non-surgical and surgical treatments exist for treatment. Like other cancers, prostate cancer has also responded to PLB treatment. PLB administration in mice harbouring PTEN-P2 tumours in the prostate induced changes in gene expression in a dihydrotestosterone (DHT)-dependent manner, suggesting that PLB activity could be mediated by androgen receptors (ARs). PLB also reduced the growth of cancer cells and induced apoptosis in DU145 and PC-3 cells [99]. The elevation of intracellular ROS by PLB results in the induction of a lethal endoplasmic reticulum stress response in Prostate Cancer (PCa) cells. 

Furthermore, PLB also demonstrated the capacity to inhibit the growth of PCa xenografts without displaying any discernible toxic effects. The administration of PLB to mice with human PCa xenografts also induced ER stress activation [100]. Like melanoma cancer cells, PLB was also found to work in synergy with other drugs for PCa. PLB was shown to significantly augment the effect of androgen deprivation therapy (ADT) drugs currently in pharmaceutical use, with only a few side effects in mice. However, a similar effect was not seen in drugs that bind to AR [101].

#### 4.1.6. Squamous-Cell Carcinoma

PLB exerts a suppressive effect on oral squamous-cell carcinoma (SCC) cells. This suppression is achieved through the induction of apoptosis, a process triggered by endoplasmic reticulum (ER) stress, ROS production, and mitochondrial dysfunction. In both drug-resistant (CAL27/RE) and drug-sensitive (CAL27) SCC cells, PLB stimulates apoptosis and autophagy by activating the JNK and AKT/mTOR pathways through ROS-mediated mechanisms. Notably, the effect on drug-resistant cells is more pronounced [102,103,104].

PLB also demonstrated a protective role by inducing autophagy and inhibiting EMT via the ROS/p38MAPK pathway in tongue squamous-cell carcinoma (TSCC) cells. In vitro studies have shown that PLB administration results in a reduction in cell viability and colony formation, accompanied by an increase in cell apoptosis. This is linked to the downregulation of GLUT1, a glucose transporter, which is mediated by the inhibition of the PI3K/Akt pathway. Studies have demonstrated that PLB can suppress tumour growth, which is correlated with the downregulation of GLUT1, in comparison to the control group [105].

Furthermore, PLB exhibits a synergistic effect when combined with existing cancer drugs. For instance, the combination of PLB and cisplatin effectively inhibits the growth of TSCC. Additionally, the combination of cisplatin, 5-fluorouracil (PF), and PLB has been reported to induce cell cycle arrest in the S phase, enhance cisplatin-induced cytotoxicity, autophagy, and apoptosis in both CAL27 and CAL27/CDDP (cisplatin-resistant) cells, and to result in higher apoptosis rates compared to PF treatment alone.

PLB has notably increased the sensitivity of TSCC cells to PF. This enhancement is achieved by inducing cell cycle arrest in the S phase and apoptosis through the PI3K/AKT/mTOR/p70S6K pathway [106]. Additionally, PLB has been demonstrated to enhance the radiosensitivity of TSCC cells. When combined with irradiation (IR), PLB leads to cell cycle arrest in the G2/M phase and, eventually, apoptosis, accompanied by the downregulation of ATM and NF-κB.

In SCC25 cells, PLB induces the G2/M-phase cell cycle arrest, inhibits EMT and stemness, and promotes extrinsic apoptosis. Notably, PLB suppresses the translocation of Nrf2 from the cytosol to the nucleus, thereby inhibiting the expression levels of downstream targets [107]. PLB has been reported to promote autophagy and cellular apoptosis in TSCC cells. This process involves the p38 MAPK and PI3K/Akt/mTOR pathways, with additional contributions from the ROS- and GSK3β-mediated pathways.

#### 4.1.7. Colorectal Cancer

Colorectal cancer (CRC) is the third most common cancer, with a five-year survival rate of less than 20% [108]. PLB has shown its anticancer properties in CRC by targeting signalling pathways affected in cancer. Alteration in miR-22-3p levels by PLB induces apoptosis and inhibits Wnt signalling and colony formation in CRC cells. MiR-22-3p plays a significant role in apoptosis, autophagy, etc., and is down-regulated in several cancers. PLB also downregulates Wnt signalling in HCT116p53+/+ and HCT116p53−/− CRC cells in a p53-independent manner and upregulates HBP1, a negative modulator of Wnt signalling in these cells [37,109,110,111]. CRC samples have shown an upregulation in expression levels of neoplastic MAPK1 and PARP1 mRNAs and downregulation in EP300 mRNA levels, whereas PLB-treated CRC cells showed results otherwise, with suppressed cell proliferation [112]. 

PLB activates the AMPK/ASK1/TRAF2 association, leading to the activation of the pro-apoptotic c-Jun N-terminal kinases (JNK)-p53 signalling axis. Following PLB treatment, activated AMPK directly leads to phosphorylation of Raptor, inhibiting the activation of mTOR complex 1 (mTORC1) and Bcl-2 expression in colon cancer cells. Furthermore, the exogenous addition of short-chain ceramide (C6) has been proven to enhance PLB-induced AMPK activation, thereby facilitating cell apoptosis and growth inhibition. The PLB-induced apoptosis of colonic cancer cells is dependent on the TNF-α-mediated pathway, with the effect being contingent on the expression of COX-2 [113].

#### 4.1.8. Pancreatic Cancer

Pancreatic cancer (PC) is a carcinoma of the pancreatic duct cells. Despite being relatively uncommon, it has a very high mortality rate [114]. PLB has shown potential inhibitory effects on PANC-1 pancreatic cancer cells. Pre-treatment with PLB significantly prevented EMT, EGF-induced survival, matrix protein hyaluron production, clonogenesis, migration, and MMP-2 gene expression and secretion in PANC-1 cells [115]. PLB significantly inhibited cell growth and induced ROS-mediated apoptosis through an intrinsic pathway, leading to the Bax and Bcl-2 being upregulated and downregulated, respectively. Additionally, PLB demonstrated an anti-migratory effect against PANC-1 cells, suggesting its potential as an antimetastatic agent [116]. Four main pharmacological targets regulated by PLB include TP53, MAPK1, BCL2, and IL6. The potential biological mechanisms involved include the advanced glycation end-product (AGE)-receptor for the advanced glycation end-product (RAGE) signalling pathway, the PI3K/Akt signalling pathway, and the hypoxia-inducible factor 1 (HIF-1) signalling pathway. These pathways play a pivotal role in regulating the survival of a cell, apoptosis, and metabolism [117]. In another study, HT in combination with PLB induced more PANC02 cell deaths than HT or PLB alone. The combination therapy demonstrated efficacy in inhibiting the accumulation of MDSCs, while simultaneously promoting the infiltration of CD4+ and CD8+ T cells within the tumour microenvironment [118].

#### 4.1.9. Cervical Cancer

Cervical cancer is one of the primary causes of cancer deaths in females across the globe, and the most frequently occurring cancer in India [119]. PLB administration demonstrated a reduction in the viability of CaSki cells in a dose-dependent manner and suppressed the migratory potential of these cells. This was attributed to reduced expression of MMP-2 and an upregulation of TIMP2. It also led to downregulation in the expression levels of E2F1 and an upregulation in the levels of p21. PLB downregulated UHRF1 expression, enhanced apoptosis, and reduced the metastasizing potential of CaSki cells. PLB also caused strong G2/M-phase arrest in CaSki and SiHa cells and S-G2/M-phase cell cycle arrest in HeLa cells [120,121]. 

PLB treatment also decreases mitochondrial membrane potential, eventually decreasing the ATP in cervical cancer cells [57,120]. PLB has been demonstrated to attenuate the HA-CD44 pathway in HeLa cells, thereby exerting an inhibitory effect on tumour growth. [122]. PLB treatment causes DNA cleavage in SiHa cancer cells. The translocation of LC-3B protein from cytoplasm to autophagosome and morphological analysis showed that PLB treatment causes autophagic cell death [123]. PLB also induced paraptosis via inhibition of the proteasome and the disruption of sulfhydryl homeostasis in cervical cancer cells (HeLa) [57]. In SiHa and HeLa cells, PLB strongly induced ROS-mediated apoptosis. At non-cytotoxic doses, PLB possesses an anti-metastatic effect, accompanied by inhibition of EMT [121]. PLB interacts synergistically with cisplatin, reducing its IC_50_ value with improved effectivity in CaSki cells [120]. 

#### 4.1.10. Ovarian Cancer

PLB treatment inhibited the growth of cancer cells and induced apoptosis in these cells. Furthermore, a reduction in the mRNA levels of intracellular OCT4 and PCNA was observed in PLB-treated cancer cells, accompanied by an increase in KLF4 mRNA activation [124]. Network pharmacology analysis revealed that PLB’s anti-UEC/COVID-19 effects are achieved via a multifaceted approach, encompassing anti-proliferation, cytotoxicity, apoptosis induction, anti-inflammatory activity, and modulation of key molecular pathways associated with anti-inflammatory activity and immunomodulation. Molecular docking studies further supported these findings by identifying potential anti-UCEC/COVID-19 pharmacological biotargets of PLB, such as mitogen-activated protein kinase 3 (MAPK3), urokinase-type plasminogen activator (PLAU), and tumour necrosis factor (TNF) [125].

#### 4.1.11. Leukaemia

The term leukaemia encompasses a diverse range of cancers, with the specific type depending on the type of blood cell affected and the rate of growth. A newly developed PLB dimer was observed to inhibit the growth of NALM-6 cells (human B cell precursor leukaemia). PLB augmented TRAIL-induced apoptosis in Kasumi-1 cells, concomitant with mitochondrial damage, up-regulation of death receptors (DRs), caspase activation, and decreased expression of cFLIP. The combination of PLB and rsTRAIL in both in vivo and in vitro models has been demonstrated to induce apoptosis of leukemic Kasumi-1 cells. Furthermore, each agent has been shown to elicit this effect independently. ROS Scavenger NAC could partially abolish the impact of PLB on the expression of Bax, DR5, and cFLIP. Conversely, glutathione (GSH) depletion by PLB was observed to increase the production of ROS [126]. PLB exerted a selective inhibitory effect on TrxR, resulting in the production of ROS in human promyelocytic leukaemia HL-60 cells. This, in turn, gives rise to an elevation of the GSSG/GSH ratio and a reduction in the cellular thiol pool [127]. The inhibition of c-Myb by PLB has been observed to suppress Myb target genes and induce differentiation of myeloid leukaemia cell line HL60 [128]. 

PLB demonstrated enhanced efficacy in the inhibition of Chronic lymphocytic leukaemia (CLL) cell lines in a dose-dependent manner. PLB also promoted the accumulation of MEC-1 cells in the S phase of the cell cycle and blocked the cell cycle transition of HG3 cells in G0/G1 to the S phase. PLB induced the apoptosis of CLL cells by increasing Bax protein expression and reducing Bcl-2 expression [129]. PLB induced a caspase-dependent apoptotic mechanism in the T-cell acute lymphoblastic leukaemia (T-ALL) cell line, MOLT-4, without any significant cytotoxicity observed in normal peripheral blood mononuclear cells (PBMCs). Furthermore, PLB has been shown to inhibit LPS-induced phosphorylation of p65 and the transcription of NF-κB target genes [130]. 

#### 4.1.12. Brain Cancer

PLB has demonstrated inhibitory effects on glioma growth in both in vitro and in vivo models. These effects are ascribed to the drug’s ability to target NQO1/GPX4-mediated ferroptosis. Specifically, PLB downregulates xCT and GPX4 while enhancing NQO1 activity, leading to NQO1-mediated cell death. Additionally, PLB promotes the degradation of GPX4 via the lysosomal pathway, resulting in GPX4-dependent cell death [131]. In nude mice with glioma cell xenografts, PLB treatment significantly reduced the tumour volume, by 54.48%. Furthermore, PLB downregulated FOXM1, along with its downstream targets (cyclin D1 and Cdc25B), while concurrently increasing the expression of p21 and p27. PLB-induced DNA damage, apoptosis, and cell cycle arrest, subsequently suppressing the ability of brain tumour cells to form a colony. This treatment also upregulated PTEN and TNFRSF1A, while downregulating E2F1 and reducing survivin, MDM2, cyclin B1, and BCL2 levels. Additionally, PLB increased caspase-3/7 activity and inhibited telomerase activity in brain tumour cells [132]. 

Studies revealed that PLB increases the transcriptional activity and nuclear localization of Nrf2, resulting in an increase in Nrf2/ARE-dependent genes in human neuroblastoma cells. PLB exposure has been shown to protect neuroblastoma cells and primary cortical neurons against oxidative and metabolic insults [133]. PLB administration significantly reduces brain damage and alleviates associated neurological deficits in a mouse model of focal ischemic stroke. PLB also directly interacts with Nox-4, inhibiting its activity in a time- and dosage-dependent manner in LN229 and HEK293 cell lines [134]. 

#### 4.1.13. Oesophageal Cancer

PLB treatment suppressed the oesophageal squamous-cell carcinoma (ESCC) cell viability and proliferation. The PLB treatment increased the proportion of cells present in the G0/G1 phase, accompanied by a corresponding decrease in the number of cells present in the S phase. PLB also targeted STAT3 signalling and inhibited ESCC cell growth [135]. PLB has been demonstrated to inhibit growth and induce apoptosis in human ESCC cells through the regulation of STAT3-PLK1-AKT signalling [136].

### 4.2. Neurodegenerative Diseases

Neurodegenerative diseases are characterized by loss of neuronal functioning, and they represent a substantial contributor to mortality and disability [137,138]. Protein kinases are considered substantial regulators for important signalling pathways, and their aberrant expression is associated with various types of cancers, metabolic disorders, and neurodegenerative disorders [139,140,141,142]. Thus, they represent optimal candidates for the development of novel therapeutic strategies. One such kinase, known as Microtubule affinity-regulating kinase (MARK4), is an isoform of the MARK family [143,144]. It regulates the phosphorylation of tau protein at specific serine residues in the microtubule-binding domain. MARK 4 also plays a role in modulating different biological functions, such as glucose homeostasis, and diseases, such as breast cancer and neurodegeneration. Computational and spectroscopic methods to analyse the binding mechanism between PLB and MARK4 showed strong binding affinity at 10^6^, as estimated. A simulation study using All-atom molecular dynamics also showed a strong binding affinity, with few conformational changes [145].

#### 4.2.1. Parkinson’s Disease

Parkinson’s disease (PD) represents the second most prevalent neurological disorder, following AD. However, only 2% of individuals aged 65 and above are afflicted with this disease [146]. Two main pathogenic aspects of this disease include the gradual deterioration of dopaminergic neurons in the substantia nigra pars compacta (SNpc) and the formation of Lewy bodies, which are primarily composed of comprise α-synuclein [147,148]. However, it is known that major pathological conditions associated with neurological diseases like depression, AD, and PD are caused by neuroinflammation [149]. Microglia cells control NF-κB activity through toll-like receptors (TLRs), which are reported to have a significant role in mediating neuroinflammation. Nuclear factor erythroid 2-related factor 2 (Nrf 2) is a transcription factor that inhibits NF-κB activity by stabilizing NF-κB inhibitor (NF-κBi) [150,151]. 

In SD rats, it is shown that PLB treatment reduces NF-κB levels and downregulates the expression of inflammatory proteins such as COX-2. It was also shown to increase the expression levels of Nrf2. The research also demonstrated that in the case of subacute and chronic PD mice models, PLB effectively inhibits inflammatory pathway activation via the TLR/NF-κB pathway and decreases the expression of TNF-α, IL-6, and IL-1*β*mRNA, thus providing neuroprotection. Furthermore, in LPS-stimulated RAW 264.7 cells, PLB has also been demonstrated to elicit an anti-inflammatory effect by downregulating pro-inflammatory mediators via the inhibition of NF-κB and MAPK signalling [19].

In addition to neuroinflammation, autophagy is a crucial target for PD treatment. The accumulation of damaged organelles and abnormal proteins is a characteristic of PD, thus pointing to a possible dysregulation of autophagy. Autophagy is primarily modulated by the mammalian target of rapamycin (mTOR) in macrophages, and has become the most significant signalling system. PLB exerts its inhibitory effect on the Akt signalling pathway by blocking Akt activation, which in turn reduces the phosphorylation of downstream targets of mTOR. 

Furthermore, it has been demonstrated that this agent reduces the level of p-mTOR protein in the substantia nigra of PD mouse models and facilitates the removal of autophagy substrate p62. Consequently, it can be concluded that this agent acts as a neuroprotective agent. Nevertheless, to substantiate PLB’s anti-PD efficacy more rigorously, additional animal and cellular PD models could be employed for future investigations [152]. PLB caused significant downregulation in the levels of various cytokines, including IL-1α, IL-12 p40/p70, IL-1β, G-CSF, MCP-1 IL-6, IL-6, MCP-5, and TNF-α [152,153].

#### 4.2.2. Alzheimer’s Disease

Alzheimer’s disease (AD) is a common neurological disorder that progresses to dementia in later stages [154]. Pathologically, AD is characterized by the accumulation of amyloidal protein deposits when β-secretase and γ-secretase act on amyloid precursor protein, which leads to the formation of β-amyloid peptide (Aβ) plaques [155,156]. AD pathogenesis is characterized by oxidative stress and subsequent neuronal damage. Usually, the antioxidant system can effectively manage oxidative stress in the cells. However, a significant increase in oxidative stress exhausts the cellular antioxidant defence mechanisms, leading to increased inflammation and subsequent neurotoxicity. Treatment with PLB in AD individuals showed that it restored the cellular antioxidant system and successfully reduced overall oxidative stress. PLB also enhances the expression of Nrf2, along with its downstream targets, HO-1 and NQO-1, in AD, to treat neuronal inflammation [157].

#### 4.2.3. Amyloid Aggregation

Amyloid aggregation of soluble proteins is one of the key factors that can cause the onset of various neuronal disorders [158]. The formation of amyloid fibrils from different proteins, including insulin and bovine serum albumin (BSA), can result in multiple medical complications. For instance, there is a documented risk of insulin aggregation into cytotoxic amyloid fibrils, especially in diabetes patients. Repeated use of insulin injections and insulin inhalers can lead to insulin aggregates in these patients. In vitro studies have also revealed the inverse association between Aβ_25–35_ positivity and serum albumin cross-linkage [159]. PLB was found to effectively inhibit insulin and serum albumin aggregation under in vitro conditions. In addition to preventing aggregate formation, it effectively promoted the disassembly of existing protein amyloids. PLB also disrupts and inhibits Human islet amyloid polypeptide (amylin) assembly into amyloid fibrils. Docking studies performed between insulin and PLB revealed strong interactions. Therefore, PLB-based formulations can be designed for such proteins, thereby preventing potential amyloid-related health risks [160,161]. Significant release of β-secretase enzyme leads to overproduction of amyloid-β. Docking studies have indicated the potential for PLB to exert an inhibitory effect on the enzyme β-secretase [162]. 

#### 4.2.4. Depression

Depression is a psychiatric disorder caused due to increased oxidative damage and monoamine neurotransmitter imbalance. The monoamine hypothesis posits that the depletion of monoamines, including serotonin, norepinephrine, and dopamine, in specific regions of the brain, including the limbic system, frontal cortex, and hippocampus, can contribute to the development of depression [163]. Monoamine oxidase (MAO) is an important enzyme involved in the metabolism of monoamines. PLB has demonstrated antidepressant-like activity in both unstressed and unstressed mice. These effects may be attributed to its ability to inhibit brain MAO-A activity and enhance the antioxidant status, as well as its capacity to elevate corticosterone levels in response to stress, reversibly [164].

### 4.3. Cardiovascular Diseases

#### 4.3.1. Hypertension

Hypertension is regarded as a potential risk factor and a major contributor to cardiovascular diseases, including heart failure, myocardial infarction, stroke, etc. Elevated cardiac output and increased vascular resistance are some characteristics of hypertension [165]. Disturbances in the regulatory mechanisms and normal compensatory mechanisms of blood pressure, including an imbalance in the retention of sodium during the excretion process, result in endothelial dysfunction and vasoconstriction, leading to hypertension. PLB is known to possess cardioprotective properties. A study by [166] investigated the role of PLB in lowering blood pressure using an invasive blood pressure (IBP) apparatus. Other parameters, viz., vascular tension and cardiac depressant effects, were monitored by performing experiments on isolated rats. PLB demonstrated the ability to reduce blood pressure, which may be attributed to a reduction in vascular resistance through the action of calcium antagonists, interference with calcium efflux, and the exertion of depressive effects on the force and rate of cardiac contraction.

#### 4.3.2. Coronary Heart Diseases

Coronary heart diseases (CHDs) contribute significantly to morbidity and mortality rates globally, and thus result in socioeconomic burden [167]. Patients suffering from Acute Myocardial infarction (MI) (a subtype of CHD) may also face severe complications such as angina pectoris and, in the worst cases, heart failure in later stages of life. Significantly reduced body weight, as well as heart weight, is observed in such patients [168]. Doxorubicin is a potent drug administered during the treatment of various cancers. However, it is highly cardiotoxic and stimulates inflammatory responses in cardiac tissues [169]. Experiments to determine the effect of PLB in doxorubicin-affected animals showed that elevated cardiac markers associated with damage of the heart and inflammatory markers were significantly reduced following PLB treatment. The PLB treatment also alleviated the overexpression of proinflammatory proteins such as NF-κB, TNF-α, and IL-1β and apoptotic proteins in the heart tissues of doxorubicin-treated rats. PLB supplementation resulted in a considerable gain in body weight and heart weight of the animals investigated. The histopathological analysis revealed that the PLB exhibited a notable protective effect on cardiac tissues against damage induced by doxorubicin [170].

#### 4.3.3. Myocardial Ischemia-Reperfusion

The role of PLB as an anti-inflammatory agent in cardiovascular diseases is further supported by its role in Myocardial Ischemia-Reperfusion (MI/R) injury, which leads to prolonged oxidative stress, loss of membrane phospholipids and endothelial dysfunction [171,172]. PLB reduced oxidative stress by decreasing lipid peroxide levels and ROS levels in rats with MI/R injury. PLB modulated the redox imbalance induced by MI/R injury by modulating the expression of transcription factors NF-κB and Nrf-2, eventually reducing the expression levels of their downstream targets. Furthermore, the expression levels of pro-inflammatory cytokines were markedly decreased by PLB treatment [173].

#### 4.3.4. Pulmonary Arterial Hypertension

Chronic inflammation leads to the development of another significant cardiovascular disease known as Pulmonary Arterial Hypertension (PAH). The disease is clinically characterized by the aggressive proliferation and resistance to cell death of the pulmonary artery smooth muscle cells (PASMCs) [174,175]. The PAH-associated phenotype is primarily attributed to the expression of STAT3 and NFAT. STAT3 activation positively regulates Pim1, the NFAT activator, leading to increased expression levels of NFAT [176]. As in cancer, PLB can also successfully inhibit the progression of PAH by targeting STAT3 both in vivo and in vitro. Therefore, the STAT3/NFAT axis can be used as a therapeutic target by PLB in human PAH-PASMCs and experimental PAH rat models [177]. 

### 4.4. COVID-19

COVID-19, caused by the novel coronavirus SARS-CoV-2, has emerged as a major global health crisis, since its discovery in late 2019. SARS-CoV-2 relies on host signalling mechanisms to propagate through RNA replication and transcription [178]. Upon entering the host cell, viruses shift the oxidant–antioxidant equilibrium towards the antioxidant state induced by Nrf-2 expression to prevent the host cell from ROS cytotoxicity and to promote mitogenic activity. In addition to this, during viral infection, several host genes associated with redox mechanisms are modulated to enable viral propagation and pathogenesis [179,180]. PLB is a known ROS inducer that possesses the ability to alter redox potential. It has known antimicrobial, anti-inflammatory, antiviral, and anticancer properties. It is, therefore, conceivable that PLB could be employed in the future to combat a pandemic, thereby preventing a significant loss of life from a potential future outbreak of a disease such as that caused by the SARS-CoV-2 virus [17]. PLB was identified as a potent main protease (Mpro) inhibitor from a natural product library. Development of an optimized FRET-based HTS assay for the discovery of Mpro inhibitors could utilize PLB as the promising lead compound to generate an antiviral agent with better potential for targeting SARS-CoV-2 Mpro [181].

### 4.5. Tuberculosis

Tuberculosis is caused by *Mycobacterium tuberculosis*, which claims nearly 2 million lives yearly, worldwide. India is among the 22 countries with an exceptionally high tuberculosis burden. It accounts for about 40% of the total number of patients afflicted with TB worldwide [182]. The alarming increase in the rate of MDR-TB cases and increased chances of HIV co-infection in such patients underscores the urgent necessity to enhance the pharmacological regimen employed for the treatment of tuberculosis. A study investigating the therapeutic potential of quinoids against tuberculosis infection showed that PLB exhibited marked inhibitory effects on the growth of *Mycobacterium tuberculosis*. Consequently, it may be regarded as a promising candidate for the development of anti-TB drugs for the management of MDR and XDR tuberculosis [20,21]. 

However, its mode of action still needs to be elucidated. It has recently been discovered that PLB mediates its anti-TB response by targeting the enzyme thymidylate synthase (ThyX), which is responsible for synthesizing dTMP from dUMP. ThyX is found selectively in pathogenic bacteria like *Mtb*, and is an essential enzyme for their survival. PLB inhibits the activity of ThyX and causes cell death by disrupting the intracellular [dTTP]/[dATP] ratio [183]. 

PLB effectively alters the intracellular redox potential in *Mtb*, causing oxidative stress [184]. Earlier studies have shown iron to be crucial for the survival of mycobacterial species. Peroxidase activity has been demonstrated to be highly sensitive to iron limitation, with a significant decrease observed in the presence of low iron concentrations [185]. A reduction in peroxidase activity results in the inability of isoniazid, an anti-Mtb drug, to undergo the necessary activation process [186]. In a docking study, a novel PLB-Isoniazid Analogue (PLIHZ) was observed to exhibit MIC values of 0.5 and 2.0 μg/mL under high and low iron conditions. These findings suggest that combining PLB with INH may prove an advantageous strategy for overcoming resistance. The cyclodextrin conjugate β-cyclodextrin inclusion complex (PLIHZCD) offers improved aqueous solubility and thermal stability, which are advantages in the treatment protocol [187]. 

### 4.6. Diabetes

Diabetes mellitus is an endocrine disorder that is characterized by hyperglycaemia, which is caused by a deficiency in insulin secretion, insulin resistance, or a combination of both. Insulin stimulates the uptake of glucose from the circulation into muscle and fat tissues via the stimulation of glucose transporter subtype 4 (GLUT 4), a member of the glucose transporter family predominantly expressed in the skeletal muscle, heart, and adipose tissues. In diabetes, as a consequence of the lack of insulin, GLUT4 is not translocated from the internal membrane to the plasma membrane, rendering GLUT4 ineffective. Thus, decreased glucose uptake by these cells results in elevated glucose levels in the blood. PLB enhanced the protein and mRNA levels of GLUT4 in diabetic rats. 

PLB also enhanced the translocation of GLUT4 and thus maintained glucose homeostasis. It also significantly reduced blood glucose levels and altered all other biochemical parameters to near-normal in STZ-induced diabetic rats. Further, PLB significantly increased hexokinase activity and decreased the activities of glucose-6-phosphatase and fructose-1,6-bisphosphatase in treated diabetic rats. The findings suggest that PLB may warrant further investigation as a potential therapeutic agent for the management of diabetes [188].

Increased glucose concentration, i.e., hyperglycaemia, leads to decreased collagen production, reduced chemotaxis, and adverse effects on wound healing [189]. Treatment with PLB in diabetic rats showed elevated levels of serum insulin, collagen deposition, and antioxidant status. PLB-treated mice showed increased protein content compared to the control, resulting in increased collagen content. High lipid levels in the blood further characterize diabetes, due to reduced levels of lipoprotein lipase in the blood. Diabetes causes the levels of total cholesterol, triglycerides, and LDL to increase and lowers the levels of HDL. Treatment with PLB significantly decreased lipid peroxides and lipid levels and increased HDL levels in the blood. Nrf2 plays an important role in wound healing. In hyperglycaemic conditions, dysfunction of keap1 leads to a decline in the levels of nrf2. This leads to impaired redox homeostasis and delayed wound healing. In diabetic mice, PLB is found to upregulate nrf2 expression levels and decrease keap1 mRNA levels. Thus, PLB administration could serve as a potent antidiabetic agent [190].

### 4.7. Other Diseases

In addition, PLB also plays a vital role as a therapeutic agent in diseases like malaria and obesity. A study on chloroquine-resistant and sensitive P. falciparum demonstrated that PLB administered at 25 mg/kg body weight for 4 days exhibits safe but low-intensity antimalarial efficacy. Thus, chemical formulations derived from the parent compound could help improve its bioavailability. In a study to investigate the role of PLB in obesity and NAFLD in mice, it was found that PLB treatment for 8 weeks improved insulin resistance and dyslipidaemia, and the mice showed a significant reduction in their body weight and obesity. This could be due to its anti-inflammatory and antioxidant properties and ability to suppress de novo lipogenesis and promote fat oxidation. 

## 5. Anti-Oxidant Activity and Anti-Inflammatory Activity of H_2_O_2_

ROS, at optimum levels, is known to support cell proliferation. Still, at increased levels, it increases DNA damage and mutagenesis in cancer cells, a possible mechanism PLB uses to exert its effect. PLB is a highly potent ROS inducer that shifts the host redox potential [17]. PLB was observed to induce DNA damage and apoptosis in cells of diverse mutational backgrounds, with comparable potency [191].

Oxidative stress is significantly associated with almost all inflammatory conditions, certain cancers, aging, neurological disorders, etc. Oxidative stress is defined as an imbalance between the production of ROS and their elimination by protective mechanisms. Oxidative stress can lead to the activation of various transcription factors, causing differential expression of some genes involved in inflammatory pathways, eventually leading to chronic inflammation. The inflammation resulting from oxidative stress can lead to many chronic diseases [192]. ROS at optimum levels is known to support cell proliferation, but at increased levels it increases DNA damage and mutagenesis in cancer cells, a possible mechanism used by PLB to exert its effect.

PLB is a potent ROS inducer that acts by shifting the host redox potential. It induces DNA damage and apoptosis in cells with diverse mutational backgrounds. Despite its role as a ROS inducer, PLB also exhibits antioxidant, anti-inflammatory, and anticancer properties [17]. In PC12 cells, PLB increased cell viability against H_2_O_2_-induced cell death by reducing oxidative stress and activating p-Nrf-2 levels. Additionally, PLB demonstrated anti-inflammatory effects by suppressing and activating NF-κB p65, downregulating the expression of COX-2 [191]. 

PLB also modulated inflammatory cytokine expression in response to H_2_O_2_-induced neurotoxic effects. It exhibited hepatoprotective activity by dampening HMGB1 expression. In a study on murine schistosomiasis, PLB treatment significantly reduced cytokine levels, restored hepatic enzyme activity, and increased antioxidant levels [193]. PLB led to a reduction in MDA levels while increasing SOD and GSH-PX levels. It also downregulated NOX4 mRNA, procollagen I mRNA, and the protein expression of NOX4 and p-IκB, as well as decreased NF-κB transcriptional activity in liver fibrosis rats [194]. 

PLB lowered the expression levels of pro-inflammatory markers, indicating its potential as a therapeutic agent for neurodegenerative diseases. In H_2_O_2_-induced chondrocytes, PLB significantly reduced oxidative stress, modulated redox and inflammation regulation transcription factors, and enhanced antioxidant defences [195]. It also demonstrated anti-inflammatory effects by downregulating COX-2, iNOS, and pro-inflammatory cytokines [153]. 

These findings suggest a protective role for PLB against H_2_O_2_-induced oxidative stress and inflammation by modulating redox signalling transcription factors. In NPCs, PLB increased viability and reduced ROS production, lipid peroxidation, and pro-inflammatory cytokine levels while elevating GSH levels and enhancing antioxidant enzyme activities. It inhibited caspase-9 and caspase-3 activity, downregulated NF-κB, and upregulated Nrf-2 expression [196], and, in addition, the neuroprotective effects of PLB in NPCs by mitigating H_2_O_2_-induced oxidative stress, inflammation, and apoptosis through the regulation of NF-κB and Nrf-2 expression. PLB also modulates redox imbalance induced by I/R injury by regulating these transcription factors and their downstream targets. Despite its chemotoxic mechanism, PLB’s activation of Nrf2 enables it to act as a chemopreventive agent.

## 6. Formulations and Binding Partners

Pharmaceutical formulation is a multi-step process. In this process, the active drug is mixed with all other components according to particle size, polymorphism, pH, and solubility, resulting in the final beneficial medicinal product. Novel formulations of PLB include liposomes, liposomes, microspheres, nanoparticles, micelles, metal nanoparticles, crystal modification, etc. Table 3 provides the list of formulations of PLB developed for therapeutic purposes.

### 6.1. Micelles

PLB release studies demonstrated a sustained-release pattern in PLB-loaded micelles (M-PLB). These micelles exhibited a higher drug release rate in acidic conditions compared to neutral conditions. In vivo, the M-PLB against *P. berghei* showed an eight-fold increase in anti-plasmodial activity compared to free PLB at the tested dosage level on the seventh day. These findings suggest that PCL-PEG-PCL micelles are promising carriers for PLB in malaria-targeting applications [197]. Tween^®^ 80 micelles also demonstrated sustained release of PLB. These micelles caused a two-fold enhancement in in vitro antitumor activity of PLB towards MCF-7 cells. The micelles were safe for intravenous injection, as PLB remained stable at high pH, and their size and encapsulation efficiency were retained upon dilution [198]. 

PLB into TPGS micelles without folic acid conjugates and PLB into TPGS micelles with folic acid conjugates increased PLB bioavailability 3.8- and 4.8-fold, respectively. These micelles exhibited extended circulation time, slower plasma clearance, and no evidence of blood and tissue toxicity as compared to those of free PLB. Micelles also demonstrated higher in vitro anticancer activity in folate-overexpressing human breast cancer MCF-7 cells [199].

### 6.2. Liposomes and Nano-Liposomes

PLB has shown good antitumor efficacy as a long-lasting circulating liposome with no evidence of normal tissue toxicity [201]. Co-delivery of PLB with PTEN plasmids in nanoliposomes (Lipo-PTEN-Plum) causes G2/M cell-cycle-arrest DNA damage and inhibits PI3K/AKT pathway, leading to apoptosis in hepatic cancer cells [200]. Another novel nano-liposomal formulation, containing PLB and the genistein drug, synergistically inhibits xenograft prostate tumour growth by ∼80% without any significant toxicity, as well as decreasing the number of Glut-1 transporters for retarding tumour growth [203]. 

PLB entrapped in transferrin-bearing liposomes resulted in increased PLB uptake by cancer cells. This enhanced uptake led to improved antiproliferative and apoptotic activity in B16-F10, A431, and T98G cell lines, compared to the drug solution. In vivo, intravenous injection of PLB-encapsulated transferrin-loaded liposomes resulted in tumour suppression in 10% of B16-F10 tumours and tumour regression in a further 10%, without any evidence of toxicity [202]. 

A nanoliposomal-based formulation of PLB with celecoxib, called CelePlum-777, was found to be stable and released these drugs in an optimal ratio for maximum synergistic killing of melanoma cells over normal cells. This formulation inhibited xenograft melanoma tumour growth by up to 72%, without any evident toxicity. The drug reduced levels of key cyclins involved in cancer cell proliferation and survival, a phenomenon that was not observed with the individual agents [201]. PLB-loaded glycerosome gel-treated rat skin showed significantly higher drug accumulation in the dermis, higher cytotoxicity, and higher antioxidant activity compared to conventional liposome gel and PLB suspension [204]. 

### 6.3. Nanoemulsions

A self-nanoemulsifying drug delivery technique improved the solubility and oral bioavailability of PLB. A study confirmed enhanced activity compared to pure PLB. A pharmacokinetic study in rats showed that a solid self-nanoemulsifying drug delivery system had more than 4-fold higher bioavailability than that of PLB alone. An ex vivo permeation study revealed that the self-nanoemulsifying drug delivery system had almost twice the intestinal permeability as that of pure PLB [205]. Novel nanoemulsion formulation of PLB based on Capryol 90 and oleic acid has shown high drug loading capacity and enhanced cytotoxicity against prostate cancer cells, PTEN-P2, compared to free PLB [23,24]. 

### 6.4. Nanoparticles

PLB-loaded Bovine Serum Albumin nanoparticles enhanced the bioavailability and decreased the toxicity of the hydrophobic drug PLB. In in silico studies, stable binding interactions between PLB and BSA are observed. BSA@PLB-NPs showed potential cytotoxicity against breast cancer cells [211]. Enhanced internalization of PLB into the HeLa cells was observed in PLB-AgNPs. PLB inhibited the proliferation of cells in a concentration-dependent manner. PLB was also observed to inhibit the clonogenic survival of cells following drug exposure, and to induce apoptosis. Furthermore, the antiproliferative, antimitotic, and apoptotic activities were enhanced when cells were treated with PLB AgNPs [195]. The intravenous administration of transferrin-loaded lipid–polymer hybrid nanoparticles loaded with PLB resulted in the disappearance of 40% of B16-F10 tumours and the regression of 10% of tumours. The mice exhibited no adverse reactions to the treatment. [212].

### 6.5. Niosomes

PLB-loaded niosomes (P-Ns-Opt) reveal a controlled release system and potential antidiabetic activity by inhibiting oxygen radicals, α-amylase, and α-glucosidase enzymes [207].

### 6.6. Microspheres

The results of the pharmacokinetic studies demonstrated a 22.2-fold increase in the elimination half-life (t(1/2)) of PLB from chitosan microspheres, in comparison to free PLB. The administration of PLB microspheres was observed to result in significant inhibition of tumour growth and a reduction in systemic toxicity [208].

### 6.7. Metal Complexes

Studies show that the Cu-PLB complex has increased cell specificity, better pharmacokinetic profile, and increased cytotoxicity. The copper complex of PLB (Cu-PLB) shows antiproliferative activity in human breast cancer cells (MCF-7) with an IC-50 of 2.3 ± 0.1, stronger than PLB alone (8.2 ± 0.2), as well as Cisplatin, a widely used anticancer drug. Cu-PLB inhibits the proliferation of HeLa, MCF-7, and murine melanoma (B16F10) cells with half-maximal inhibitory concentrations (IC50), lower than those achieved with PLB alone. Cu-PLB caused microtubule disassembly, induction of ROS, and DNA damage. In breast cancer, Cu-PLB induces apoptosis through MAPK-mediated inhibition of anti-apoptotic protein Mcl-1, thereby inhibiting cancer progression [209,213]. Four copper(II)-plumbagin and -bipyridine complexes (Cu1-Cu4) showed enhanced anti-tumour activity by accumulation in mitochondria, causing their dysfunction, activating caspase-9/3, and inducing apoptosis of cancer cells [210].

## 7. Limitations and Challenges

Like many phytochemicals, PLB exhibits both pro-oxidant and antioxidant properties, necessitating careful consideration of its effects on cells and tissues, particularly with respect to dosage and in vitro factors such as pH, media composition, oxidative stress, and oxygen amounts. These factors can reveal unexpected biological effects by uncovering hidden mechanisms of action [31]. Therefore, before any clinical application, it is crucial to identify bioavailable and safe doses that minimize risks for patients. Further chemical modifications of PLB may enable targeted specificity for particular cell types, or enhance its bioavailability for in vivo studies.

However, PLB faces several challenges that hinder its clinical translation, including poor water solubility (79 μg/mL), high lipophilicity (log P 3.04), unstable nature (spontaneous sublimation), and low oral bioavailability (less than 40%). Additionally, its therapeutic concentration in tumours is difficult to achieve, due to its low specificity and rapid elimination [202]. PLB accelerated xanthine oxidation in mouse liver S9 (MLS9), human liver S9 (HLS9), and XO monoenzyme system. PLB was shown to be well-bound to XO. In addition, in vivo studies have demonstrated that PLB significantly increased serum uric acid levels and enriched serum XO activity in mice [214]. Despite being a potential bioactive molecule, PLB still finds limited use in medicine. While many formulations have provided a solution to overcome the limitations, further research will help put PLB on the market as an efficient drug.

## 8. Conclusions and Prospects

PLB, a member of the naphthoquinones, has been broadly utilized as conventional medicine, owing to its various health benefits. Through multiple studies, it has been demonstrated as a potential therapeutic drug for diseases including cancer, neurodegenerative disorders, genetic disorders, and lifestyle diseases. PLB has been reported to play a significant role in preventing a broad range of malignancies, including cancers with high mortality rates, such as breast cancer, lung cancer, etc. PLB has shown promising results via its anti-proliferative, anti-angiogenesis, anti-metastatic activities, and induction of apoptosis in cancer cells. 

Several studies have demonstrated that when PLB is used in combination with other drugs, it displays enhanced antitumor effects, reinforcing its potential in cancer therapy. The therapeutic roles of PLB are not limited to cancer. The versatility of its medicinal properties lies in its antioxidant and anti-inflammatory nature. PLB has been found to play curative and restorative roles in neurodegenerative diseases, genetic diseases, lifestyle diseases, and viral and bacterial diseases. 

Despite its benefits and cytoprotective activity in various diseases, PLB remains in the cradle of drug research. Its poor pharmacokinetics limit human use. Low bioavailability, lack of specificity, and some reports on toxicity in mice have restricted the promotion of PLB as an industrially important drug. Various strategies have been implemented to overcome these limitations, such as nanoparticles, emulsions, metal complexes, etc. These formulations have ensured more efficient and safer delivery of PLB with enhanced therapeutic power. 

PLB represents a novel anti-tumour drug with the potential to yield promising results in both pre-clinical and clinical trials. Therefore, future research should concentrate on laboratory studies, with the aim of expanding in-depth research on the molecular mechanism of PLB and developing a strategy for creating potent formulations that minimize toxicity and maximize efficacy.

## Figures and Tables

**Table 1:** Chemical properties and drug-like features of Plumbagin.

Properties	Values
Molecular Weight	188.18
XLogP3	2.3
Log P	1.72
Hydrogen-Bond Donors	1
Hydrogen-Bond Acceptors	3
Rotatable Bonds	0
Topological Polar Surface Area	54.4 Å^2^
Heavy Atoms	14
Formal Charge	0
Complexity	317
Isotope Atoms	0
λmax	
Melting point	77
Boiling point	383.927
**Drug-like features**	
Log Po/w	1.72
GI absorption	Yes, very high 96.258
BBB Permeability	Yes
Skin Permeability	No, −2.933
Caco2 permeability	Low, 1.192
Water Solubility	High, −2.655
Bioavailability score	0.55

**Table 2:** The anticancer effects of plumbagin have been reported in different cell lines.

S. No.	Disease	Cell Line	IC_50_ (nM)	Potential Mechanism(s)	Reference
1	Breast Cancer	MCF7	2840	Cytotoxic agent against human MCF7 cells expressing HER2 that showed reduced cell viability after 48 h via CellTiter-Glo assay	[32]
2	Breast cancer	MDA-MB-468	2500	Role as an antiproliferative agent against ER-negative human MDA-MB-468 cells after 48 h by MTT assay	[33]
3	Breast Cancer	MDA-MB-231	3500	Reduced proliferation of ER-negative human MDA-MB-231 after 24 h analysed by MTT assay	[33]
4	Ductal breast carcinoma	BT-474	800	Cytotoxic for Her2-overexpressing human BT474 cells after 72 h analysed by MTT assay	[34]
5	Melanoma	SK-MEL-28	5000	Inhibited the growth of human SK-MEL-28 cells analysed by MTT assay	[35]
6	Colorectal cancer	HCT-116	9800	Cytotoxic for human HCT116 cells by inhibiting growth	[36]
7	Colon adenocarcinoma	SW480 SW-620	7300 7400	Cytotoxic role assessed by cell viability	[37]
8	Colorectal cancer	HT-29	4190	Cytotoxic against human HT-29 cells	[38]
9	Hepatocellular carcinoma	HepG2	9170	Cytotoxic against human HepG2 cells	[36]
10	Lung carcinoma	A549	3000	Inhibited growth of human A549 cells	[35]
11	Leukaemia	HL-60	1100	Cytotoxic against human HL60 cells; assessed reduced cell viability in cells	[39]
12	-	PBMC	2700	Cytotoxic against human PBMC cells	[39]
13	Cervical cancer	HeLa	10,200	Cytotoxicity	[40]

**Table 3:** Plumbagin formulations.

S. No.	Category	Name of Formulation	Effect on the Therapeutic Role of PLB	Disease	Reference
1	Micelle	PLB-loaded micelles (M-PLB) PCL-PEG-PCL	There was an eightfold increase in anti-plasmodial activity.	Malaria	[197]
		Tween^®^ 80	Sustained release of PLB, enhanced antitumor activity.	Breast Cancer	[198]
		PTM & PTEM	Increased bioavailability and circulation, no blood toxicity.	Breast Cancer	[199]
2	Liposomes	Lipo-PTEN-Plum nanoliposomes	Restoration of PTEN, G2/M cell cycle arrest, and cell death via inhibition of PI3K/AKT pathway.	Hepatocellular carcinoma	[200]
		PLB-loaded long circulating pegylated liposomes	No tissue toxicity.	Cancer	[201]
		Transferrin-bearing liposomes	Increased uptake, improved antiproliferative and apoptotic activity.	Cancer	[202]
		PLB and genistein	Inhibits tumour growth by ~80%.	Prostate cancer	[203]
		CelePlum-777	Stable release, decreased levels of key cyclins.	Melanoma	[95]
		Glycerosome	Deeper skin-layer penetration, higher drug accumulation.	Skin cancer	[204]
3	Nano-emulsion	Self-emulsifying drug-delivery system.	Higher bioavailability,	Anti-inflammatory	[205]
		Capryol 90-based and Oleic-acid-based nanoemulsion.	high drug-loading capacity with enhanced cytotoxicity.	Prostate cancer	[23]
4	Nanoparticles	BSA@PLB-NPs	Cytotoxicity against cancer cells.	Breast cancer	
		PLB-AgNPs	Enhanced internalization, antimitotic and antiproliferative.	Breast Cancer	[206]
		Plumbagin Entrapped in Transferrin-Conjugated, Lipid–Polymer Hybrid Nanoparticles	Disappearance, along with regression, of tumour in mice.	Melanoma	[80]
5	Niosome	P-Ns-Opt	Controlled release inhibits oxygen radicals, α-amylase, and α-glucosidase enzymes.	Diabetes.	[207]
6	Microspheres	Chitosan microspheres	Increase in elimination half-life of PLB.	Melanoma	[208]
7	Metal complex	Cu-PLB	Increased cell specificity and cytotoxicity, induction of ROS, and DNA damage.	Breast Cancer	[209]
		Cu1-Cu4	Mitochondria dysfunction, and apoptosis, cell cycle arrest at S phase.	Cervical carcinoma	[210]

## Data Availability

All data generated or analysed during this study are included in this manuscript.
